# Critical Excitation of the Fundamental Quasi-Shear Mode Wave in Waveguide Bars for Elevated Temperature Applications

**DOI:** 10.3390/s19040793

**Published:** 2019-02-15

**Authors:** Jiuhong Jia, Zuoyu Liao, Xiaotao Cai, Yun Tu, Shan-Tung Tu

**Affiliations:** Key Laboratory of Pressure Systems and Safety, Ministry of Education, East China University of Science and Technology, Shanghai 200237, China; siogexyu@163.com (Z.L.); xiaotao_cai@outlook.com (X.C.); ytu@ecust.edu.cn (Y.T.); sttu@ecust.edu.cn (S.-T.T.)

**Keywords:** guided wave, fundamental shear horizontal wave, waveguide bar, anti-plane shear line source, nondestructive testing

## Abstract

The safety of critical pressure equipment in elevated temperature is increasingly important. Moreover, the on-line monitoring method is potentially useful to improve their safety. A waveguide bar system can enable monitoring of critical equipment working in elevated temperature using reliable ultrasonic technology. Among the waveguide bar system, the matching mechanism of the transducer and the waveguide bar is crucial to propagate the pure fundamental quasi-shear mode (shorten for SH0*) wave. In the present research, the loading line sources that can excite pure SH0* wave are investigated and the anti-plane shear loading source is selected. The critical values about the geometric dimensions of the junctions between the piezoelectric transducer and the waveguide bar are explored by simulation and experiments. On the condition that the excitation sources satisfy the critical values, the loading can be approximated to an anti-plane shear one to excite the pure SH0* wave. Some waveguide bar systems are designed based on the simulated critical values and some experiments at high temperature are carried out. The experimental results verify that the designed waveguide bar systems can excite the pure SH0* wave at elevated temperatures, which verify the reliability of the simulated critical results.

## 1. Introduction

Pressure equipment is the critical component in process and energy industries. The initiation and propagation of cracks in the equipment is a fatal factor inducing major accidents [1,2,3,4]. The guided wave technology has been studied as an effective tool to nondestructively detect cracks [5,6,7,8,9]. Therefore, introducing guided wave technology to on-line monitor these components is a potential useful way to ensure the health of the pressure equipment [10,11]. However, when the ultrasonic transducer is installed long-termly on elevated temperature equipment for on-line monitoring, the transducers will depolarize, which limits its application in high temperature [12].

In order to apply guided wave technology in elevated temperature successfully, the waveguide technology has already been pursued by many researchers [13,14,15,16,17]. Because the waveguide act as a signal transmission medium between the high-temperature specimen and the piezoelectric transducers, the fragile piezoelectric transducer can be isolated from the high-temperature component to ensure the transducer to work long-termly. During the development of waveguides, the bars with different cross sections have been analyzed recently [13,14]. Among these waveguide bars, rectangular cross-section ones have been successfully applied in several cases [15,16,17]. Therefore, the research about rectangular waveguides are increasingly popular. Fan et al. have demonstrated the existence of nonlinear guided waves in an aluminum plate and a steel rectangular bar [18]. Rymantas et al. have investigated a family of SH-types which can propagate in the case of finite-width waveguides [19]. Cawley et al. have derived the criteria for rectangular bar to transmit some fundamental waves [20].

Because of the characteristics of the fundamental shear horizontal (shorten for SH0) wave that it is non-dispersive, and it is less affected by the presence of surrounding media demonstrate, it is potentially useful for on-line monitoring applications [21,22,23]. Strictly speaking, the term “fundamental shear horizontal” doesn’t make sense in a waveguide bar other than an infinite plate. Hence the fundamental quasi-shear mode (shorten for SH0*) is chosen to indicate the fundamental shear horizontal mode in a waveguide bar. The excitation of the SH0*. wave has been discussed in some research. The geometrical criteria of SS304 plates with large aspect ratio to excite the non-dispersive SH0* wave have been developed by Cegla et al. [24,25]. The critical values of the 316L stainless plate to transmit the nondestructive SH0* wave have been calculated by Jia et al. [26]. This research focuses on avoiding wave dispersion and scattering when waves go through waveguide bars. However, the main challenges to improve signal quality in the waveguide bar system are the dispersive nature of wave propagation at the transducer/waveguide junction. The matching mechanism between transducer and waveguide bar will affect both the energy and the wave modes propagating through the waveguide bar for elevated temperature applications, which have not been rigorously studied.

Therefore, in order to excite the non-dispersive SH0* wave for the elevated temperature applications, matching relationships between transducers and waveguide bars are studied in the present research.

## 2. Excitation Characteristics Analyses

**Figure 1:**
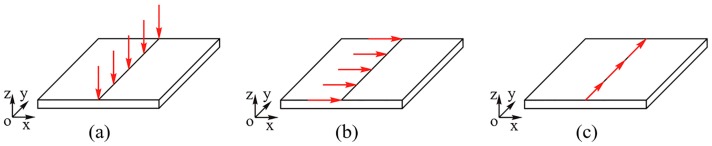
The typical line source loading types: (**a**) Normal loading; (**b**) Tangential shear loading; (**c**) Anti-plane shear loading.

The junction between the transducer and the waveguide bar is called as the excitation source. The cross section of the bar with large aspect ratio can be taken as a line. That is to say, the excitation of the transducer for the bar is an approximate line loading. The line source loading types generally includes normal loading, tangential shear loading and anti-plane shear loading [25]. Different loadings show different excitation abilities, as shown in Figure 1.

**Figure 2:**
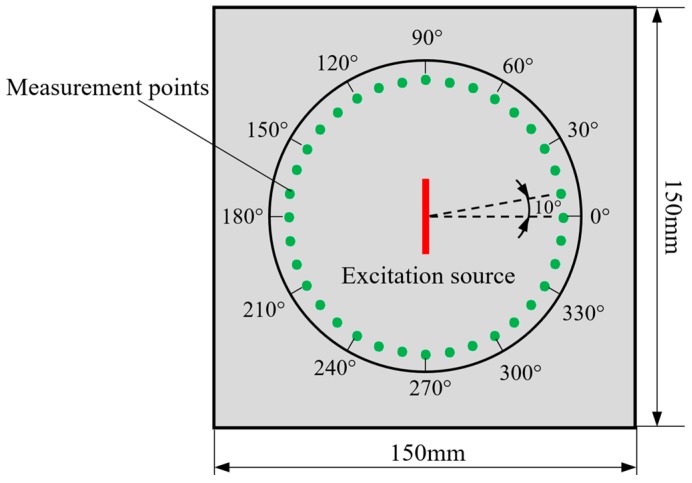
Layout of the line source and measurement points.

In order to select an appropriate loading to excite the non-dispersive SH0 wave, the finite element simulation of three loading types are carried out using ANSYS software. A 316L stainless steel rectangular plate is applied as a specimen. Its dimensions are 150 mm × 150 mm × 1 mm. The material parameters are Young’s modulus *E* = 195 GPa, Poisson’s ratio *υ* = 0.267 and density *ρ* = 2700 kg/m^3^. The line source lies in the middle of the plate and is supposed as a rectangle section in dimensions of 20 mm × 0.2 mm.The Plate is modeled using the Solid 164 element. The maximum size of the elements is 0.2 mm and the time step is selected as 0.05 μs. The excitation signal is a 5-cycle sinusoidal displacement tone burst modulated by a Hanning window and the center frequency *f*_c_ is 1 MHz. The displacement load is applied according to the three loading conditions mentioned above, respectively. The displacement signals are measured at every 10° to obtain the distribution of the guided waves on the surface of the plate, as shown in Figure 2.

**Figure 3:**
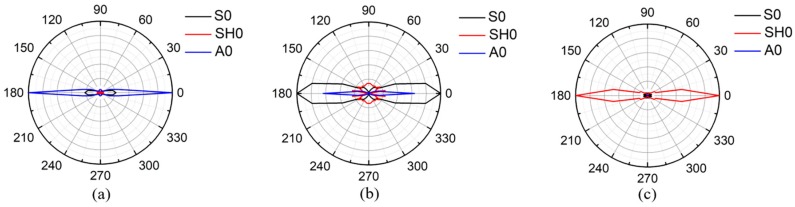
Directivity of guide waves excited by different types of line sources on plates: (**a**) Normal loading; (**b**) Tangential shear loading; (**c**) Anti-plane shear loading.

Figure 3 shows simulation results of the directivity of guided waves excited by line sources. For waves excited by the normal loading, the SH0 wave is much weaker than the A0 wave, which can be found in Figure 3a. Similarly, for waves excited by tangential shear loading, the SH0 wave is significantly weaker than the S0 wave, as shown in Figure 3b. However, for waves excited by the anti-plane shear loading, the SH0 wave is stronger than the Lamb waves. In this case, the Lamb waves can be ignored, which is plotted in Figure 3c, and the SH0 wave is perpendicular to the line source with a narrow beam. Therefore, the anti-plane shear loading shows an excellent ability to excite the SH0 wave.

Moreover, Cegla et al. have verified that the directivity of the anti-plane shear source radiates equally and strongly in all directions in the semi-infinite space [25].

Therefore, we can conclude that the anti-plane shear loading is the premium line source to excite the pure SH0 wave, based on the purity and the directivity of the SH0 wave excited by the line source of anti-plane shear loading.

## 3. Critical Excitation Analysis

**Figure 4:**
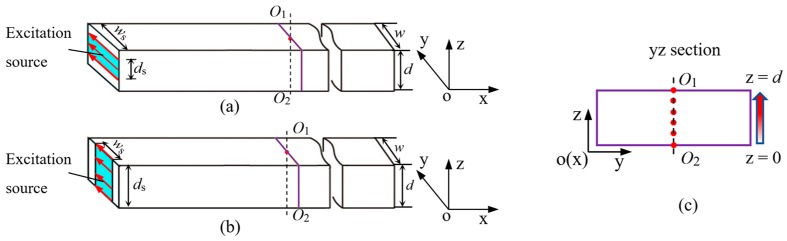
Locations of the excitation sources in the end section of the waveguide bar: (**a**) Equal width symmetric match; (**b**) Equal thickness symmetric match; (**c**) Analyzed nodes in the cross section of the waveguide bar.

The anti-plane shear loading can be easily loaded at the end of a waveguide bar [20,25]. In order to investigate whether the change of excitation source affects the waveform, the waves from the part surface of the waveguide bar are excited gradually to the whole surface. The representative loading methods are shown in Figure 4. The highlighted area denotes the excitation source. The width of a waveguide bar is *w*, and the thickness is *d*. The width of the excitation source is named as *w*_s_, and the thickness is *d*_s_.

Generally, the distributions of the anti-plane shear loading in end section of the waveguide bar can be split into two representative matching styles. Figure 4a represents an equal width symmetric match (Shorten for EWS). In this case, the excitation source is symmetrically arranged along the longitudinal centerline of the end section. *w*_s_ equal to *w*, and *d*_s_ is not bigger than *d*. *d*_s_ increases from 0.2*d* to *d* at an interval of 0.2*d* during the simulation. Figure 4b represents an equal thickness symmetric match (Shorten for ETS). The excitation source is symmetrically arranged along the transverse centerline of the end section. *d*_s_ equals to *d*, and *w*_s_ is not bigger than *w*. *w*_s_ increases from 0.2*w* to *w* at an interval of 0.2*w*.

For the simulations, four waveguide bars are designed. Their length is 100 mm and width is 25 mm. Thickness are different which are 0.5 mm, 1 mm, 6 mm and 8 mm, respectively. For the waveguide bars with thickness of 0.5 mm and 1 mm, their frequency-thickness production *fd* are 0.5 MHz⋅mm and 1 MHz⋅mm, which are smaller than the first cut-off frequency-thickness production (*fd*)_1_ is 1.6 MHz⋅mm [21]. For the waveguide bars with thickness of 6 mm and 8 mm, their *fd* are 6 MHz⋅mm and 8 MHz⋅mm which are bigger than (*fd*)_1_. Wave signals are extracted at six nodes in the central lines O_1_O_2_ of the cross section of the waveguide bar. The nodes are extracted along the thickness direction from z = 0 to z = *d* at an interval of 0.2*d*, as shown in Figure 4c. According to the wave structure of the SH0 wave, if only the pure SH0* wave are excited in waveguide bars, the received signal at each node should be consistent. On the contrary, if some higher order SH modes occur, the signals extracted from each node may be different.

**Figure 5:**
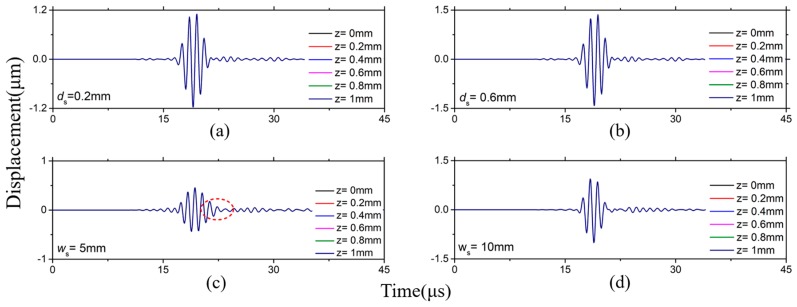
Waveforms in 1 mm thick waveguide bars: (**a**) *w*_s_ = 25 mm, *d*_s_ = 0.2 mm; (**b**) *w*_s_ = 25 mm, *d*_s_ = 0.6 mm; (**c**) *w*_s_ = 5 mm, *d*_s_ = 1 mm; (**d**) *w*_s_ = 10 mm, *d*_s_ = 1 mm.

When *fd* are smaller than (*fd*)_1_, a 1 mm-thick waveguide bar and a 0.5 mm-thick waveguide bar are analyzed. Figure 5 shows the waveform of waves excited by the excitation sources with different matching styles in a 1 mm thick waveguide bar. The wave signals excited by the EWS are shown in Figure 5a,b, and the signals excited by the ETS are depicted in Figure 5c,d. It is noticed that the signals don’t change when the thickness of the excitation source changes from 0.2 mm to 1 mm. According to the group velocity and the wave structure of SH0* wave, it is easy to know that the wave excited in the waveguide bar is the SH0* wave. This shows that no matter how thick the excitation source is, the non-dispersive SH0* wave can always be excited in the waveguide bar. And the wave slightly disperses when *w*_s_ is 5 mm, as shown in Figure 5c. Moreover, the wave dispersion weakens with the increase of the excitation source’s width and the wave dispersion can be ignored when *w*_s_ is bigger than 9 mm, as shown in Figure 5d. The same conclusion can be obtained when the simulation is carried out using a 0.5 mm thick waveguide bar. Therefore, 9 mm is a critical width value.

**Figure 6:**
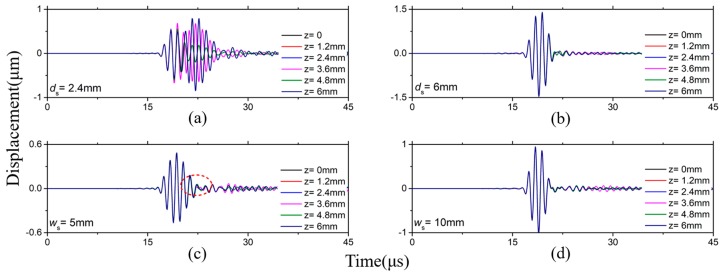
Waveforms in 6 mm thick waveguide bars: (**a**) *w*_s_ = 25 mm, *d*_s_ = 2.4 mm; (**b**) *w*_s_ = 25 mm, *d*_s_ = 6 mm; (**c**) *w*_s_ = 5 mm, *d*_s_ = 6 mm; (**d**) *w*_s_ = 10 mm, *d*_s_ = 6 mm.

When *fd* are bigger than (*fd*)_1_, let’s take the waveguide bar with a thickness of 6 mm and 8 mm as examples. Figure 6a shows that it is impossible to excite the pure SH0* wave if *d*_s_ is smaller than *d*. However, it can excite the pure SH0* wave when *d*_s_ equal to the thickness *d*, as shown in Figure 6b. Moreover, ETS can excite the pure SH0* wave when *w*_s_ is bigger than 9 mm, and the waves disperse slightly when *w*_s_ is smaller than 9 mm, as shown in Figure 6c,d. The same conclusion can be obtained when the simulation is carried out using an 8mm thick waveguide bar. Therefore, 9 mm is the critical length of excitation source, too.

When we change to different simulation frequencies, this critical value will change. If the excitation frequency *f*_c_ is 500 kHz, the critical value is 18 mm. A generally critical value is explored by a lot of simulated at different frequencies. It is 3*λ*. Therefore, when *fd* are smaller than (*fd*)_1_, the *w*_s_ of the excitation source should be wider than 3*λ*, and the thickness is free. And when *fd* are bigger than (*fd*)_1_, *w*_s_ should be wider than 3*λ* and the *d*_s_ equal to *d* [27].

## 4. High Temperature Performance Tests

### 4.1. Experimental System Setup

In order to verify the exciting of the SH0* wave, some waveguide bars are manufactured. Their length is chosen as 400 mm which is long enough for the temperature to drop to 23 °C at the distal end of the waveguide from 350 °C at the proximal end during the testing period. Their width is selected as 18 mm on the consideration of non-dispersion. Thicknesses are 0.5 mm, 1 mm, 2 mm, 3 mm and 4 mm, so that some *fd* are smaller than (*fd*)_1_ and some are bigger.

In this waveguide bar system, a d15 shear mode actuation piezoelectric transducer installed on the end of the bar execute the anti-plane shear loading line source. The cross sections of the transducer equal to the excitation source. The loading source changes with the cross section of the waveguide bar.

**Figure 7:**
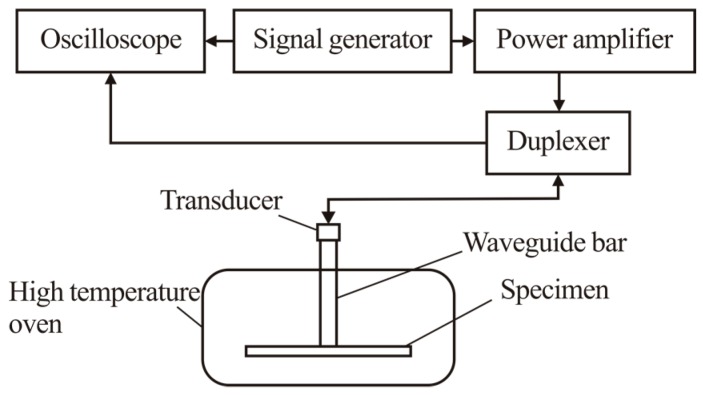
The sketch map of the experimental system.

The experimental system is setup as shown in Figure 7, which is composed of oscilloscope MDO3012 (Tektronix, INC., Beaverton, OR, USA), function generator AFG 3021C(Tektronix, INC., Beaverton, OR, USA), power amplifier AG1006 (T&C Power Conversion, INC., Rochester, NY, USA), duplexer (Ritec, INC., Warwick, RI, USA), piezoelectric transducer (Olympus V154-RM, Olympus NDT, INC., Waltham, MA, USA), waveguide bars, specimen plate, related clamps and a high temperature oven. The waveguide bars and the specimen plate are all 316L stainless steel. The dimensions of the test plate is 150 mm × 150 mm × 10 mm. The 316L stainless steel bars are attached to the specimen by a specific clamp, so that the clamping forces attached on each waveguide bar are constant. The specimen is placed in the oven. One end of the waveguide bar is mounted on the test plate and the other end passes through the thermal insulation layer staying outside of the oven.

### 4.2. Experimental Results Analyses

**Figure 8:**
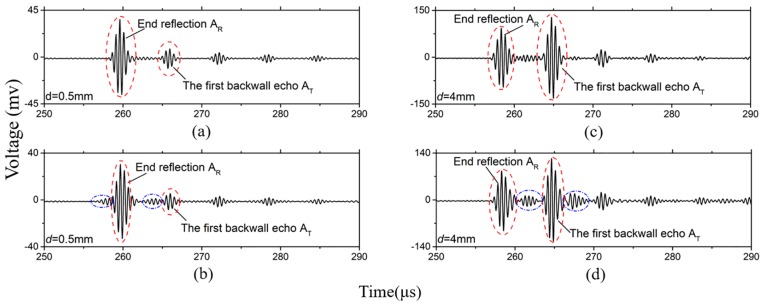
Waveforms of the time domain in some waveguide bars: (**a**) *d* = 0.5 mm, *w* = 13 mm, the loading is anti-plane shear source; (**b**) *d* = 0.5 mm, *w* = 13 mm, the loading isn’t anti-plane shear source; (**c**) *d* = 4 mm; *w* = 13 mm; the loading is anti-plane shear source (**d**) *d* = 4 mm, *w* = 13 mm, the loading isn’t anti-plane shear source.

During testing, the five-cycle tone bursts modulated by a Hanning window are generated at 2 MHz. The waves excited by these systems are shown in Figure 8. The waveforms in 0.5 mm-thick bar is plotted in Figure 8a,b, on the condition that the *fd* is smaller than (*fd*)_1_. The waveforms in a 4 mm-thick bar is plotted in Figure 8c,d, in this case that the *fd* is bigger than (*fd*)_1_. The width of the excitations source in Figure 8a is 13 mm, the thickness is 0.5 mm, the width is 13 mm and the thickness is 4 mm in Figure 8c. These waveforms are clear, and there is no dispersion. They are the SH0* mode judging by their speeds. However, excitations sources in Figure 8b,d are rotated 30° relative to its original position. That is to say, the anti-plane shear loading source is changed. In this case, the waves are dispersive, as shown in Figure 8b,d. The waveforms in the blue ellipse aren’t the SH0* wave. When we shorten the width of the anti-plane shear loading source, the waves are still dispersive. These results confirm that the anti-plane shear loading source can excite the pure SH0* wave when its geometrical structure meets the critical conditions.

Once the ultrasonic signal is excited and propagates along the waveguide bar, only a part of the wave energy can transmit into the specimen for defect detection. In order to verify this wave energy, the transmission efficiencies are compared. The transmission efficiency *η* is defined as the ratio of the amplitude A_T_ of the first echo signal and the amplitude A_R_ of the end reflection signal. The A_T_ and A_R_ are shown in Figure 8. Because the clamping forces of bars remain unchanged, so that the error from the coupling is small enough to be neglected when the transmission efficiencies are studied.

**Figure 9:**
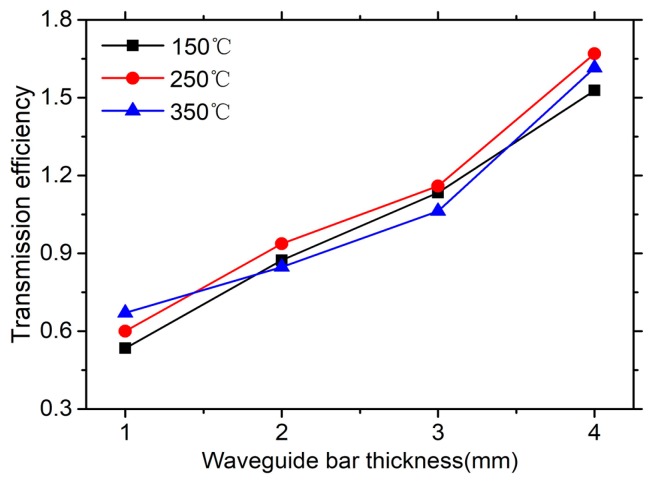
The transmission efficiency of the SH0* wave versus waveguide thicknesses.

Curves of the transmission efficiency *η* versus thickness are shown in Figure 9. They monotonically increase. These curves shows that the thicker the waveguide is, the more energy of the SH0* wave is transferred into the test plate for damage detection. Moreover, the effect of temperature change on the transmission efficiency can be neglected.

According to the special mounting style, the excitation loading source changes with the cross section of the waveguide bar. Therefore, the transmission efficiencies increase when the waveguide bars become thicker. However, the heat dissipation performance decreases when the bars become thicker. For the engineering application, the waveguide bars can design a bit thicker, on the condition that the excitation wave is non-dispersion and the heat dissipation meets requirements.

## 5. Conclusions

In order to excite the non-dispersive SH0* wave using a waveguide bar system for elevated temperature applications, the critical excitation mechanism of the SH0* wave at the junction of the PZT transducer and the waveguide bar is studied. Firstly, the characteristics of line sources to excite waves with different loading conditions are simulated. And the anti-plane shear loading line source that can excite the pure SH0* wave is verified. Secondly, the matching mechanism of the transducer and the bar is investigated. It is found that when the frequency-thickness product of the corresponding waveguide bar is less than the first cut-off frequency, the pure SH0* wave can be excited only if the length of the excitation source is greater than 3*λ*, and when the frequency-thickness product of the bar is bigger than its first cut-off frequency, the SH0* wave can be excited on the condition that the length of the excitation wave source is greater than 3*λ* and the thickness of the excitation wave source equals to that of the waveguide bar. At last, some waveguide transducers are designed and high temperature experiments are carried out. Experimental results show that the designed waveguide bar system can excite the pure SH0* wave and the excitation ability isn’t affected by high temperature. Moreover, some guidance to design the waveguide transducers for elevated temperature application are generalized.
